# Effects of Bleaching Protocols on the Esthetic Outcome and Surface Integrity of Enamel Treated With an Ammonia‐Free Silver Fluoride Solution

**DOI:** 10.1111/jerd.70136

**Published:** 2026-03-05

**Authors:** Ahmed Alshawi, Fadimeana Lal, Esra Yildiz, Sevda Ozel Yildiz, Ugur Erdemir

**Affiliations:** ^1^ Department of Restorative Dentistry Istanbul University, Faculty of Dentistry Istanbul Turkey; ^2^ Department of Biostatistics Istanbul University, Faculty of Medicine Istanbul Turkey

**Keywords:** ammonia‐free silver fluoride, color stability, enamel surface roughness, laser‐activated bleaching, teeth bleaching

## Abstract

**Objective:**

To investigate the effects of three bleaching protocols on the color stability and surface roughness of enamel previously treated with a water‐based, ammonia‐free silver fluoride solution (Riva Star Aqua).

**Materials and Methods:**

Eighty‐eight human enamel/dentin specimens were demineralized and treated with Riva Star Aqua, divided into four groups (*n* = 22): Control (no bleaching), home bleaching with 15% hydrogen peroxide (HP), in‐office bleaching with 40% HP, and laser‐activated bleaching with 46% HP. Color differences (Δ*E*_00_) were measured at different stages, and the surface roughness (Ra) was evaluated before and after bleaching. Data were analyzed using two‐way repeated measures ANOVA and Fisher's PLSD test (α = 0.05).

**Results:**

The application of Riva Star Aqua caused perceptible discoloration in all groups. Following bleaching, all three active protocols resulted in significant color correction compared with the control group (*p* < 0.05). The final color differences (Δ*E*_00_ Final) for Opalescence Boost (6.82 ± 0.49), Opalescence Home (8.84 ± 0.42), and Laser White 20 (8.83 ± 0.37) were statistically comparable (*p* < 0.05). At the same time, the control group showed significantly high Δ*E*_00_ Final (10.75 ± 0.29). Regarding surface roughness, all bleaching groups showed a significant increase in Ra values (*p* < 0.05) compared to baseline, with significant differences between Laser White 20 and the other bleaching protocols (*p* < 0.05).

**Conclusion:**

Bleaching effectively reversed the discoloration caused by ammonia‐free silver fluoride. While all tested protocols achieved comparable final color correction, the laser‐activated protocol resulted in significantly less alteration of surface roughness than the conventional in‐office and at‐home bleaching protocols.

## 1 Introduction

Dental caries is the most common non‐communicable disease in adults, affecting 2.3 billion people worldwide [1]. In adult and geriatric populations, the prevalence of root caries is particularly high and increases with age [2]. Older individuals are more vulnerable to this condition due to a distinct set of predisposing risk factors [2]. Primary among these issues are gingival recession and alveolar bone loss, which expose the sensitive root surfaces to the oral environment. Furthermore, this susceptibility is exacerbated by age‐related physiological and lifestyle changes, including limited manual dexterity, the use of removable prostheses, and dietary shifts toward simple sugars [2]. Crucially, salivary flow is frequently compromised in this demographic, often manifesting as xerostomia induced by polypharmacy and systemic conditions, which significantly reduces the natural protective buffering capacity against cariogenic challenges [3].

Historically, the management of dental caries was dominated by G.V. Black's philosophy of “extension for prevention,” which prioritized the mechanical retention of restorations often at the expense of sound tooth structure. However, a deeper understanding of caries as a dynamic, biofilm‐driven disease process, along with major advances in adhesive technologies, has led to a fundamental shift in perspective. The current surgical restorative approach to treating dental caries is widely practiced but faces challenges regarding long‐term sustainability and cost‐effectiveness [4]. This approach often involves a repetitive “restorative cycle” in which restorations fail over time. Consequently, this leads to a gradual loss of tooth structure and increased costs [2]. Studies indicate that direct restorations have annual failure rates ranging from 1% to 5%, primarily due to secondary caries at the restoration margins [5].

Modern dentistry has shifted from an aggressive surgical approach to a “medical model” focused on disease management. This current philosophy emphasizes early detection, risk evaluation, and preserving as much natural tooth structure as possible using minimally invasive and non‐surgical approaches [6].

Therefore, caries treatment protocols worldwide have shifted from a surgical‐restorative model to a minimally invasive, non‐surgical approach. In this method, only the outer infected dentin is removed, while therapeutic agents are applied to arrest the lesion and preserve the inner affected dentin [7]. These include sodium fluoride (NaF) [8] and casein phosphopeptide‐amorphous calcium phosphate (CPP‐ACP) [9], which promote remineralization, as well as chlorhexidine (CHX) [10], which is used for its broad‐spectrum antimicrobial activity and ability to inhibit matrix metalloproteinases [4].

Given the proven efficacy of topical fluoride in preventing dental caries [4], it has been hypothesized that a combination of silver and fluoride not only stops existing caries but also prevents the development of new caries [8]. There are two chemical forms of silver‐fluoride combinations: silver fluoride and silver diamine fluoride (SDF) [11]. In the past, 40% silver fluoride was used in Western Australia to prevent tooth decay in children [9]. Although silver fluoride (AgF) is less commonly available today, silver diamine fluoride (SDF, 38% w/v) has become the predominant silver‐fluoride‐based agent used in clinical practice [10]. The potential of SDF as a caries‐preventive treatment is supported by studies showing it is more effective than fluoride varnish. SDF has emerged as an affordable, non‐invasive option for stopping and preventing dental caries, with research backing its clinical performance over other treatments [3, 10]. SDF is a complex of silver, fluoride and ammonia; the ammonia acts as a ligand, forming a diamine silver complex, which increases chemical stability [12]. This coordination with ammonia inhibits the quick precipitation of silver, which is a common instability in simple silver fluoride (AgF) solutions, thus enabling SDF to preserve its ionic concentration [13].

The well‐documented antimicrobial efficacy of silver salts, demonstrated during their historical use in medicine and dentistry, is an essential component of the mechanism of SDF [14]. The applications of SDF in dental care are diverse and include the treatment of caries in young children and the prevention of root caries in older adults. SDF is also used to prevent pit and fissure caries and secondary caries and to reduce tooth sensitivity [15]. Recent studies suggest that it also helps in the treatment of infected root canals and the strengthening of teeth that have undergone endodontic treatment [16]. The various effects of SDF solutions support their classification as bioactive materials [17]. These effects include the purported reduction of cariogenic bacterial growth and biofilm formation [14], occlusion of dentinal tubules [18], promotion of tertiary dentin formation [19], and the dual effect of inhibiting the demineralization of dentin while promoting its remineralization [14].

The main disadvantage of SDF as an anti‐caries treatment is the noticeable dark discoloration on the tooth surface it contacts [20, 21]. This discoloration occurs because the residual silver ions react with organic material [22]. In a recent survey of adult patients, a significant minority (21.3%) opposed the use of SDF in the anterior region and a further 25.3% were strongly opposed, mainly due to esthetic concerns [23]. Several strategies have been proposed to reduce tooth discoloration caused by SDF application. These include the sequential use of potassium iodide (KI) after SDF [24], the incorporation of KI directly into the SDF formulation [25], the modification of SDF with the glutathione bio‐molecule (GSH) [26] and the exploration of silver nanoparticles as a less discoloring alternative to conventional SDF [27].

The efficacy of these proposed strategies to reduce SDF‐mediated staining is inconsistent, showing varying success rates and contradictory results [28]. These conflicting outcomes are often attributed to high heterogeneity across studies, including variations in methodology, the specific silver agents tested (e.g., concentrations and additives), differing application protocols, and inconsistent quantification of color change.

At the same time, a new water‐based, ammonia‐free 38% silver fluoride solution (Riva Star Aqua, SDI, Melbourne, Australia) has recently been launched [29]. The manufacturer claims that this water‐based formula overcomes ammonia‐based SDF products. However, these claims rely mainly on manufacturer information and require independent scientific validation. They also state that the product is less irritating to soft tissue, has a milder odor, and offers better storage stability [29]. However, as it is new to the market, there is currently no data on its effects on tooth color or its potential to cause discoloration. While data on this new ammonia‐free formulation is absent, the esthetic challenge of traditional SDF staining is well documented. Several in vitro studies have investigated the use of bleaching protocols to reverse ammonia‐based SDF stains, with varying success. Some studies found that at‐home carbamide peroxide bleaching significantly lightened discoloration [30]. Others have explored in‐office bleaching, suggesting it may also be a viable option [21]. Nevertheless, this data relates to stains induced by ammonia‐based SDF, and it remains unclear whether a water‐based, ammonia‐free silver fluoride solution will result in staining of the enamel surface, or if these protocols will be effective against stains if such stains are already present due to a water‐based, ammonia‐free formulation.

A concurrent and essential concern across all bleaching protocols is their potential to compromise the integrity of the enamel surface [31, 32, 33]. High‐concentration in‐office agents, in particular, have been shown in some studies to increase surface roughness (Ra) and porosity, which negatively impact the long‐term integrity of the tooth [34, 35, 36, 37, 38]. It is unknown how an enamel surface pre‐treated and remineralized with a novel water‐based, ammonia‐free silver fluoride agent would respond to this chemical challenge.

Therefore, the aim of this in vitro study was to investigate the effects of different bleaching protocols on the color stability and surface roughness of enamel remineralized with this water‐based, ammonia‐free silver fluoride agent.

Based on the pre‐existing knowledge from related materials, the research hypotheses tested were:

1. The water‐based, ammonia‐free silver fluoride solution will cause a clinically perceptible color change in demineralized enamel.
2. The application of different bleaching protocols will significantly lighten the enamel discoloration caused by the water‐based, ammonia‐free silver fluoride treatment.
3. The application of bleaching protocols will significantly increase the surface roughness of the treated enamel.

## 2 Materials and Methods

### 2.1 Determination of the Specimen Size

The specimen size was determined in advance using G*Power 3.1.9.7 software (Heinrich Heine University, Dusseldorf, Germany) based on an F‐test (ANOVA). The power analysis was conducted considering both main outcome variables: color difference Δ*E*_00_ and surface roughness (Ra). The analysis indicated that a total sample size of 88 specimens (22 per group) was sufficient to detect a medium effect size (*f* = 0.5) for both variables with a statistical power of 80% and a significance level of 0.05. The selected sample size is consistent with the specimen size reported in the previous study [26]. The same samples were used to evaluate both color stability and surface roughness.

### 2.2 Experimental Design

This in vitro study used a randomized experimental design to evaluate the effects of bleaching on enamel treated with Riva Star Aqua. The study design was organized as follows:

#### 2.2.1 Experimental Groups

Eighty‐eight specimens were randomly allocated into four distinct groups (*n* = 22):

- Group 1 (Control): No bleaching treatment.
- Group 2 (At‐Home Bleaching): Treated with 15% hydrogen peroxide (Opalescence Home, Ultradent Products, South Jordan, UT, USA).
- Group 3 (In‐Office Bleaching): Treated with 40% hydrogen peroxide (Opalescence Boost, Ultradent Products, South Jordan, UT, USA).
- Group 4 (In‐office Laser‐Activated Bleaching): Treated with 46% hydrogen peroxide activated by a diode laser (Laser White 20, Biolase Technology, San Clemente, CA).

#### 2.2.2 Dependent Variables and Measurement Time Points

- Color Differences (Δ*E*_00_): The primary outcome was measured at three specific time points:
  1. Baseline (before demineralization).
  2. After remineralization with Riva Star Aqua.
  3. After the bleaching protocol.
- Surface Roughness (Ra): The secondary outcome was measured at two time points:
  1. Baseline (immediately before the bleaching protocol).
  2. After the bleaching protocol.

#### 2.2.3 Instruments and Parameters

- Colorimetry: A portable spectrophotometer (Vita Easyshade, VITA Zahnfabrik, Bad Sackingen, Germany) was used to capture CIELab coordinates for all color measurements [39, 40].
- Profilometry: A contact profilometer (Surtronic S‐128, Taylor Hobson, Leicester, UK) was used to quantify surface roughness (Ra) in micrometers (μm) [41].

**FIGURE 1:**
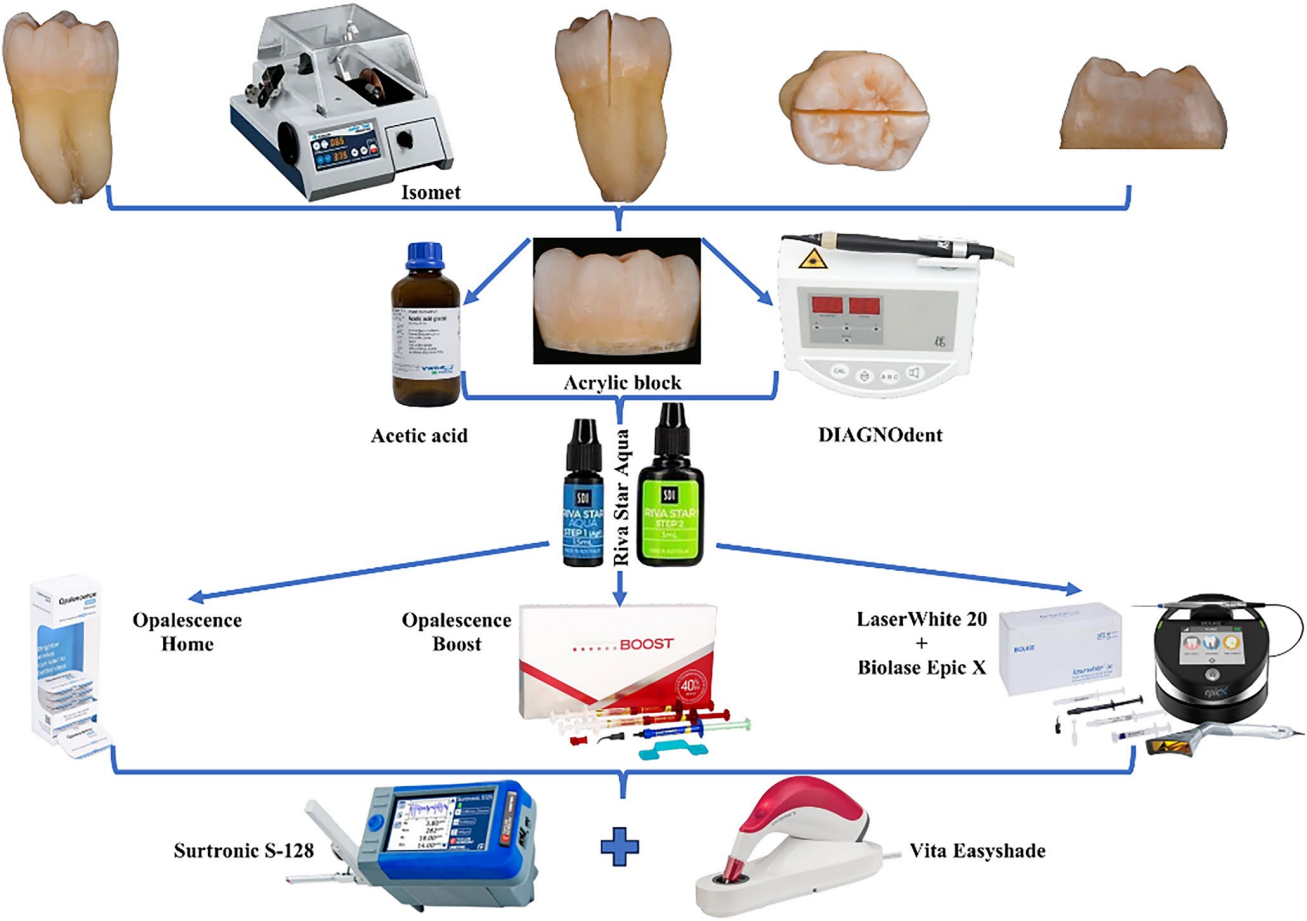
Schematic representation of the materials and equipment used in each stage of this study.

The materials and equipment used in this study are shown in Figure 1.

### 2.3 Specimens Preparation

Non‐carious, intact human permanent molars were obtained anonymously from donors who had provided written informed consent for use in the study. The study protocol was reviewed and approved by the Ethics Committee of Istanbul University Faculty of Dentistry (Protocol No. 2024/27). Enamel and dentin plates with a minimum size of 6 × 6 × 4 mm were cut from the buccal and lingual surfaces of 44 caries‐free molars using a low‐speed diamond saw (Isomet, Buehler, Lake Bluff, IL, USA) under constant water irrigation to avoid thermal damage. During the initial collection and preparation phase only, the teeth were stored in a 0.1% thymol solution to inhibit microbial growth. Residual soft tissue and plaque were removed by gentle brushing under distilled water. Each specimen was embedded in a white, self‐curing acrylic resin block (12 × 12 × 10 mm; Varidur, Buehler), with the enamel and dentin plates positioned to protrude 2 mm above the acrylic resin surface, thereby exposing the surface. Following embedding, the specimens were thoroughly rinsed to remove any residual thymol. All specimens were then stored in artificial saliva composed of 2.20 g/L gastric mucin, 1.45 mM CaCl_2_‐2H_2_O, 5.40 mM KH_2_PO_4_, 28.4 mM NaCl, and 14.9 mM KCl, adjusted to a pH of 7.0 [42]. They were kept for 24 h before baseline measurements, with the solution refreshed daily to ensure a stable environment. Throughout the entire experiment, all specimens were kept in an incubator at a constant 37°C. The initial CIELab colorimetric values were recorded three times: as a baseline after a 24‐h stabilization period, after 1 month of applying Riva Star Aqua, and 48 h following bleaching, all measured with the Vita Easyshade spectrophotometer.

### 2.4 Caries‐Like Lesion Creation (Demineralization)

Artificial carious lesions were created on the exposed tooth surfaces of each specimen with a demineralizing solution prepared by the Electrochemical Laboratory of the Chemistry Department of Istanbul University. The solution was developed according to the protocol outlined in the study [43], consisting of 2.2 mM calcium chloride (CaCl_2_), 2.2 mM sodium dihydrogen phosphate (NaH_2_PO_4_) and 0.05 mM acetic acid. The pH was adjusted to 4.4 with 1.0 M potassium hydroxide (KOH) to simulate acidic conditions that promote enamel demineralization. Each specimen was immersed in 10 mL of the prepared solution in sealed containers and kept at room temperature for 4 days. The solution was refreshed every 48 h to ensure constant demineralizing activity. Lesion progression was monitored using a DIAGNOdent device (Kavo Dental, Biberach, Germany), and specimens were considered sufficiently demineralized when a fluorescence value of 30 or higher was reached. Specimens that did not reach this threshold were returned to the demineralization solution for further exposure. After demineralization, all specimens were thoroughly rinsed with distilled water, air‐dried and stored in artificial saliva.

### 2.5 Remineralization Using Riva Star Aqua

Riva Star Aqua was applied for the surface treatment of specimens. All steps were carried out exclusively by a trained examiner (A.A.), who followed all steps meticulously and in strict accordance with the manufacturer's instructions. A single drop of Riva Star Aqua Step 1 was placed on a non‐absorbent mixing pad and applied to the enamel surface with controlled movements using a medium‐sized microbrush. Two drops of Riva Star Aqua Step 2 were then placed on a clean pad and applied with a new microbrush. The treated surface initially showed a creamy white appearance. The Step 2 application was continued until the surface became transparent, indicating the reaction was complete. The enamel was then air‐ dried to remove residual moisture. After treatment, all specimens were stored in artificial saliva, which was refreshed daily to maintain a stable environment. The specimens were incubated at 37°C for 1 month to simulate intraoral conditions. At the end of the incubation period, the second color measurement was performed with the Vita Easyshade spectrophotometer to evaluate the changes in tooth color after treatment.

### 2.6 Division of the Specimens and Formation of Groups

Following the remineralization phase, the specimens were randomly assigned to four groups, each of which underwent a different bleaching protocol, as explained in Part 2.2.1.

### 2.7 Bleaching Protocols

All bleaching materials were used according to the respective manufacturer's instructions, as shown in Table 1.

**TABLE 1:** Outlines the ingredients and protocols of the bleaching materials used in this study.

Bleaching agent	Ingredients	Time	Repetition
Opalescence Home	Glycerin, Aqua (Water), 15% Hydrogen Peroxide, Silica, PVP, Acrylates/C10‐30 Alkyl Acrylate Crosspolymer, Potassium Hydroxide, Dipotassium Phosphate, Citric Acid, Disodium EDTA, Mentha Piperita (Aroma), Potassium Nitrate, Sodium Fluoride, Sucralose	20 min	1× day for 10 days
Opalescence Boost	40% Hydrogen Peroxide, Water, Carbopol, Propylene Glycol, Glycerin, Synthetic Amorphous, Pyrogenic Silica substance, Potassium Nitrate, Potassium Hydroxide, Sodium Fluoride, Dimethicone.	20 min	3 treatments in one session
Laser White 20	46% Hydrogen Peroxide	20 min	1 treatment

**In group 1** (control) the specimens were stored in artificial saliva in an incubator at 37°C.

**In group 2** (At‐Home Bleaching), a 15% hydrogen peroxide (HP) gel (Opalescence Home), pH 6.5, was applied to the enamel surface in a 0.5–1.0 mm thick layer for 20 min daily for 10 days. After each application, the bleaching gel was wiped off with a cotton swab. The specimens were rinsed with running distilled water for 1 min to remove any residual bleach. After the bleaching protocol, specimens were stored in artificial saliva at 37°C in an incubator for 48 h.

**In group 3** (In‐Office Bleaching), a 40% hydrogen peroxide gel (Opalescence Boost) with a pH of 6.0–8.5 was used. A 0.5–1.0 mm‐thick layer was applied to the specimen's surface for 20 min and then wiped off with a cotton swab. This 20‐min application was repeated twice more during the same session, so that the treatment lasted a total of 60 min, according to the manufacturer's instructions. After the last application, the bleaching gel was wiped off with a cotton swab, and each specimen was rinsed with running distilled water for 1 min to remove any residual bleach. The specimens were then stored in artificial saliva at 37°C in an incubator for 48 h.

**In Group 4** (In‐office Laser‐Activated Bleaching) involved a single 20‐min application of 46% hydrogen peroxide (Laser White 20). A 0.5–1.0 mm thick layer of gel was applied to the surface of the preparation. The bleaching gel was activated with a Biolase Epic X 940 nm diode laser (Biolase Technology, San Clemente, CA) operated in continuous‐wave mode at 940 nm for all applications in this study [44, 45]. The bleaching handpiece, set to a fixed 7 W, was held 1–2 mm from the specimen for 30 s without touching the gel. After 20 min, the bleaching gel was wiped off with a cotton swab. The specimens were then rinsed with running distilled water for 1 min to remove residual bleach and stored in artificial saliva at 37°C in an incubator for 48 h.

### 2.8 Color Assessment

Reflectance spectra were measured using the CIELab system, and values for chroma (C*) and hue angle (h°) were calculated for each specimen at three different times: baseline, 1 month following remineralization with Riva Star Aqua, and 48 h later bleaching. A single trained operator (F.L.) performed all measurements using a Vita Easyshade spectrophotometer. A custom silicone guide was made for each specimen to ensure reproducibility and prevent ambient light or probe angulation from affecting measurements. It positioned the spectrophotometer tip consistently on the enamel surface. Each measurement was repeated three times per specimen, and the average was calculated. The color difference Δ*E*_00_ was calculated to represent the changes between stages using the following equation:

$$\Delta E_{00}=\sqrt{{\left(\frac{\Delta L^{\prime }}{k_{L}S_{L}}\right)}^{2}+{\left(\frac{\Delta C^{\prime }}{k_{C}S_{C}}\right)}^{2}+{\left(\frac{\Delta H^{\prime }}{k_{H}S_{H}}\right)}^{2}+R_{T}\left(\frac{\Delta C^{\prime }}{k_{C}S_{C}}\right)\left(\frac{\Delta H^{\prime }}{k_{H}S_{H}}\right)}$$

The Δ*E*_00_ value ≤ 0.8 indicates a difference below the perceptibility threshold (PT), meaning the color change is imperceptible to the human eye. The Δ*E*_00_ value ≤ 1.8 falls below the acceptability threshold (AT), denoting a clinically acceptable color match [46].

The color differences were measured three times with different references, as shown in Table 2.

**TABLE 2:** Color difference measurements for various references and explanations of these measurements.

1	Δ*E*_00_ Aqua	Remineralization—Baseline (Color difference between the surface treated with Riva Star Aqua and the original surface at basline)
2	Δ*E*_00_ Bleaching	Bleaching—Remineralization (Color difference between the surface treated with bleaching agents and the surface treated with Riva Star Aqua)
3	Δ*E*_00_ Final	Bleaching—Baseline (Color difference between the surface treated with bleaching agents and the original surface at basline)

### 2.9 Roughness Assessment (Ra)

Surface roughness was measured on each specimen both before and after bleaching using a mechanical contact profilometer (Surtronic S‐128, Taylor Hobson, Leicester, UK). To eliminate the interfering influence of the demineralization solution, whose erosive effects on enamel roughness are well documented, the roughness measurements were carried out immediately before bleaching and after 48 h of bleaching. The measurements were carried out using a diamond stylus with a tip radius of 5 μm, a cut‐off value of 0.80 mm, a transverse length of 0.80 mm and a scanning speed of 0.25 mm/s. A calibrated examiner (A.A.) performed profilometric measurements at three different points in the central area of each specimen to ensure consistency and reproducibility. The profilometer was calibrated before each measurement.

### 2.10 Statistical Analysis

Before statistical analysis, all data were checked for normality and homoscedasticity using the Shapiro–Wilk test and Levene's test, respectively. The color differences (Δ*E*_00_ Aqua, Δ*E*_00_ Bleaching and Δ*E*_00_ Final) and surface roughness (Ra) were analyzed using a two‐way repeated measures ANOVA, with “bleaching protocol” as the between‐subject factor and “measurement time point” as the within‐subject factor. All pairwise comparisons of ANOVA analyses were performed using Fisher's Protected Least Significant Differences (PLSD) to control the overall significance level to 5%.

## 3 Results

### 3.1 Color Stability (Δ*E*_00_ )

Table 3 presents the measured values of the CIELab system, along with the calculated chroma (C*) and hue angle (h°) for each specimen at three different times.

**TABLE 3:** Mean values (standard deviation) of tested specimens for CIELab coordinates with the chroma (C*) and hue angle (h°).

Nu	Bleaching protocol	CIELab coordinates and chroma (C*) and hue angle (h°) values				
		Before demineralization—Baseline				
		L*	a*	b*	C*	H°
1	Control Group	81.06 ± 0.22	0.01 ± 0.01	18.12 ± 0.34	18.12 ± 0.12	89.98 ± 0.37
2	Opalescence Home	81.15 ± 0.18	0.01 ± 0.01	17.85 ± 0.29	17.0.85 ± 0.10	89.98 ± 0.31
3	Opalescence Boost	80.91 ± 0.21	0.00 ± 0.01	17.93 ± 0.27	17.93 ± 0.09	90.01 ± 0.30
4	Laser White 20	81.21 ± 0.23	0.04 ± 0.01	18.24 ± 0.32	18.24 ± 0.14	89.88 ± 0.35

Nu	Bleaching protocol	After remineralization with Riva Star Aqua				
		L*	a*	b*	C*	H°
1	Control Group	72.52 ± 0.41	2.01 ± 0.08	18.03 ± 0.37	18.14 ± 0.17	83.65 ± 0.26
2	Opalescence Home	72.86 ± 0.37	1.81 ± 0.04	17.50 ± 0.31	17.59 ± 0.19	84.10 ± 0.23
3	Opalescence Boost	72.62 ± 0.39	1.97 ± 0.03	17.91 ± 0.26	18.02 ± 0.14	83.73 ± 0.27
4	Laser White 20	72.05 ± 0.4	1.74 ± 0.06	17.98 ± 0.30	18.07 ± 0.18	84.48 ± 0.29

Nu	Bleaching protocol	After bleaching				
		L*	a*	b*	C*	H°
1	Control Group	72.31 ± 0.55	1.56 ± 0.09	17.07 ± 0.92	17.17 ± 0.21	82.65 ± 0.29
2	Opalescence Home	80.41 ± 0.63	0.40 ± 0.02	9.05 ± 0.79	9.06 ± 0.24	87.48 ± 0.31
3	Opalescence Boost	80.63 ± 0.60	0.30 ± 0.01	11.14 ± 0.80	11.14 ± 0.18	87.90 ± 0.27
4	Laser White 20	78.59 ± 0.58	0.37 ± 0.02	10.02 ± 0.84	10.03 ± 0.19	87.89 ± 0.24

**FIGURE 2:**
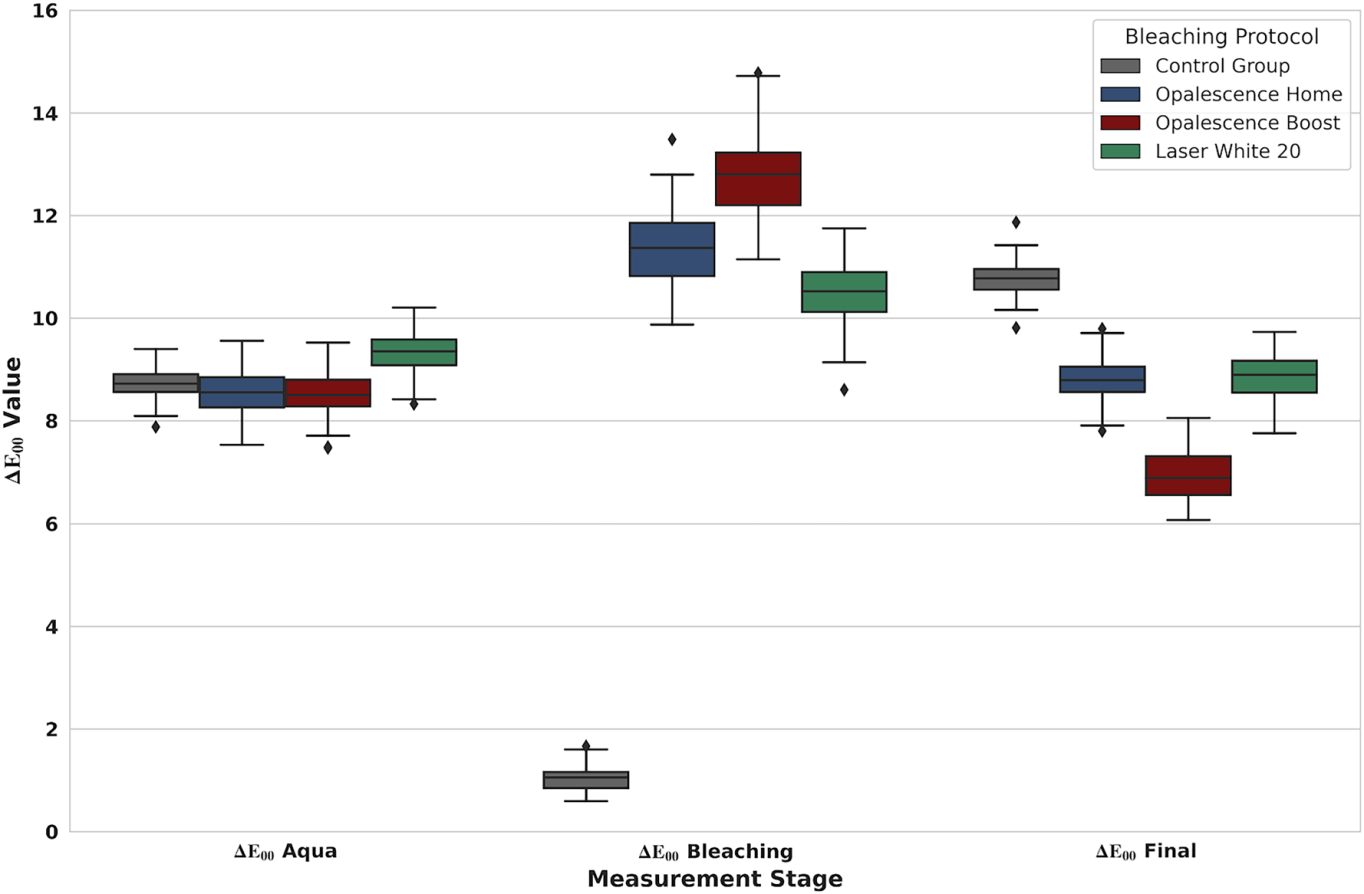
Color difference Δ*E*_00_ across experimental stages.

Color differences Δ*E*_00_ between the four test groups—Control, Opalescence Home, Opalescence Boost, and Laser White 20—were calculated at different stages. Comparisons with the mean Δ*E*_00_ values for the references specified in Table 2 are shown in Table 4 and graphically in Figure 2.

**TABLE 4:** Mean values and standard deviations of the color difference Δ*E*_00_ values.

Nu	Bleaching protocol	Δ*E*_00_		
		Δ*E*_00_ Aqua	Δ*E*_00_ Bleaching	Δ*E*_00_ Final
1	Control Group	8.77 ± 0.34^a^	1.04 ± 0.23^b^	10.75 ± 0.29^b^
2	Opalescence Home	8.53 ± 0.47^a^	11.42 ± 0.67^a^	8.84 ± 0.42^a^
3	Opalescence Boost	8.52 ± 0.39^a^	12.75 ± 0.77^a^	6.82 ± 0.49^a^
4	Laser White 20	9.32 ± 0.41^a^	10.40 ± 0.63^a^	8.83 ± 0.37^a^

*Note:* Lower case showing significant difference among bleaching protocols (column, *p* < 0.05).

#### 3.1.1 Effect of Remineralization (Δ*E*_00_ Aqua)

Following the application of Riva Star Aqua, all experimental groups exhibited a clinically perceptible color change, with Δ*E*_00_ values significantly exceeding the acceptability threshold (Δ*E*_00_ > 1.8). The mean color differences ranged from 8.52 ± 0.39 (Opalescence Boost) to 9.32 ± 0.41 (Laser White 20). Statistical analysis revealed no significant differences among the four groups at this stage Δ*E*_00_, confirming that the baseline discoloration was uniform prior to the bleaching protocols.

#### 3.1.2 Effect of Bleaching Protocols (Δ*E*_00_ Bleaching)

The bleaching phase induced significant color changes in all active treatment groups. The Opalescence Boost group showed the highest color change relative to the stained state (12.75 ± 0.77), followed by Opalescence Home (11.42 ± 0.67) and Laser White 20 (10.40 ± 0.63). There were no statistically significant differences among these three bleaching protocols (*p* > 0.05). However, all three active groups exhibited significantly higher Δ*E*_00_ values compared to the control group (1.04 ± 0.23), which showed only minor color variations attributable to aging and storage (*p* < 0.05).

#### 3.1.3 Final Color Outcome (Δ*E*_00_ Final)

The final color difference relative to the original baseline revealed significant disparities between the control and the treated groups. The control group exhibited the highest final Δ*E*_00_ value (10.75 ± 0.29), indicating that the silver‐fluoride discoloration persisted and remained significantly different from the original tooth color. In contrast, the bleaching protocols successfully reduced the total color difference, returning the enamel closer to its baseline shade. The Opalescence Boost group resulted in the lowest final color difference (6.82 ± 0.49), followed by Laser White 20 (8.83 ± 0.37) and Opalescence Home (8.84 ± 0.42). Statistical analysis indicated that all three bleaching groups were significantly more effective than the control group (*p* < 0.05), with no statistically significant differences observed among the three active protocols regarding their final esthetic outcome.

### 3.2 Surface Roughness (Ra)

**FIGURE 3:**
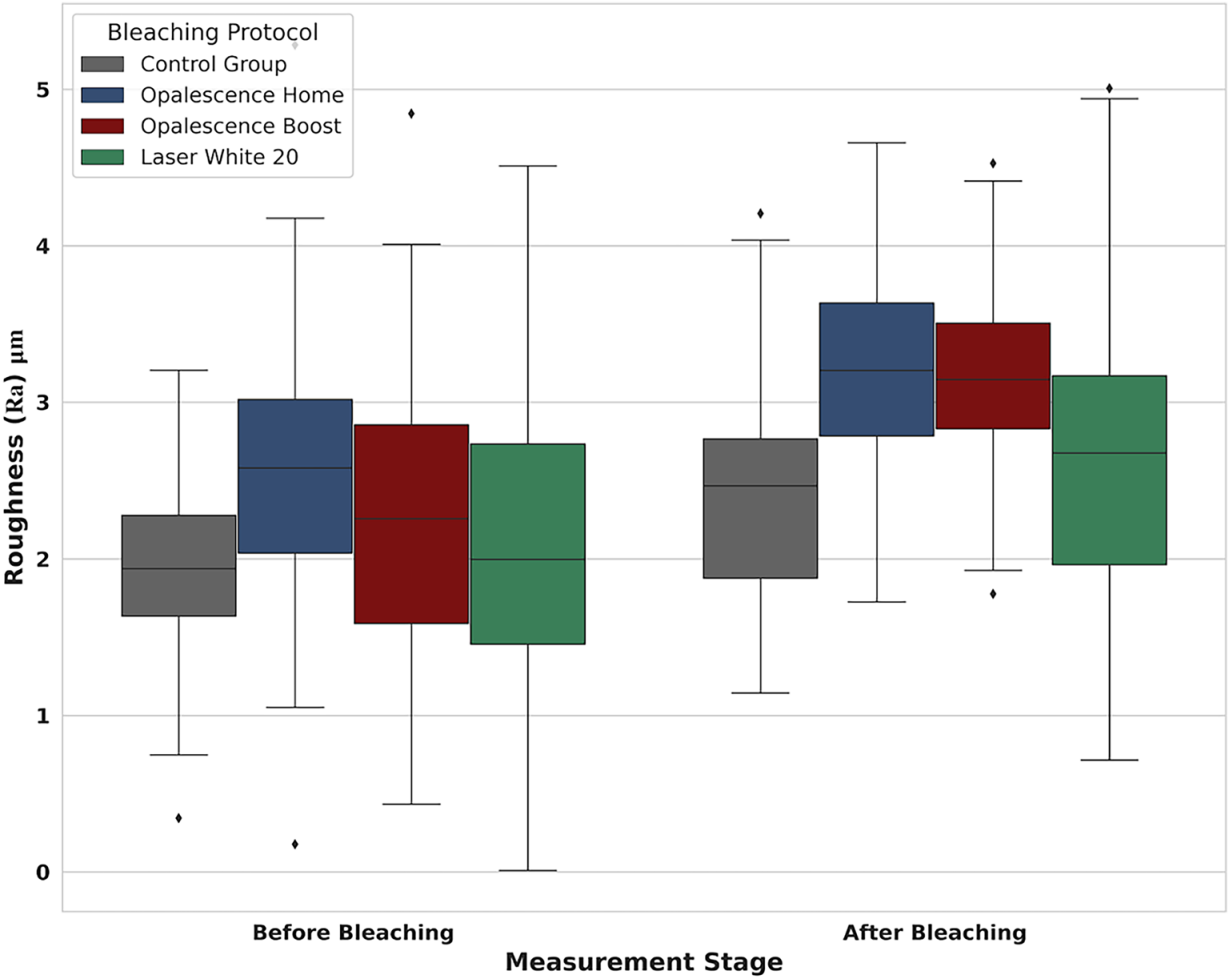
Surface roughness (Ra) before and after bleaching.

The surface roughness (Ra) of the enamel was measured before and after bleaching in each test group. Data are expressed as mean ± standard deviation and analyzed for both within‐group differences (before and after bleaching) and between‐group differences (between the different bleaching protocols), as shown in Table 5 and Figure 3.

**TABLE 5:** Surface roughness values (Ra, μm) and standard deviations of the groups.

Nu	Bleaching protocol	Roughness (Ra) μm	
		Before bleaching	After bleaching
1	Control Group	2.02 ± 0.64Aa	2.41 ± 0.66Aa
2	Opalescence Home	2.51 ± 0.72Aa	3.17 ± 0.68Bb
3	Opalescence Boost	2.32 ± 0.82Aa	3.21 ± 0.58Bb
4	Laser White 20	2.04 ± 0.96Aa	2.61 ± 0.91Ba

*Note:* Capital letters indicate significant difference before and after bleaching (row, *p* < 0.05), while lowercase letters between bleaching protocols (column, *p* < 0.05).

#### 3.2.1 Baseline Roughness (Before Bleaching)

Prior to the bleaching treatments, there were no statistically significant differences in surface roughness among the four groups (*p* > 0.05). The initial Ra values ranged from 2.02 ± 0.64 (Control) to 2.51 ± 0.72 (Opalescence Home), ensuring a standardized baseline for comparison.

#### 3.2.2 Intragroup Analysis (Effect of Time)

The longitudinal analysis (Before vs. After) revealed different patterns of surface alteration among the groups. The control group showed no statistically significant change in roughness over time (2.02 ± 0.64 vs. 2.41 ± 0.66; *p* > 0.05). In contrast, all three active bleaching protocols—Opalescence Home, Opalescence Boost, and Laser White 20—exhibited a statistically significant increase in surface roughness following treatment compared to their respective baseline values (*p* < 0.05), as indicated by the change from uppercase letter “A” to “B”.

#### 3.2.3 Intergroup Analysis (Comparison of Protocols)

Following the bleaching phase, significant differences were observed between the groups (*p* < 0.05).

- The Opalescence Home (3.17 ± 0.68) and Opalescence Boost (3.21 ± 0.58) groups exhibited the highest roughness values. Both were significantly rougher than the control group (*p* < 0.05).
- Notably, while the Laser White 20 group showed an increase from its own baseline, its final roughness value (2.61 ± 0.91) was statistically comparable to the control group (2.41 ± 0.66; *p* > 0.05) and significantly lower than both the Opalescence Home and Opalescence Boost groups (*p* < 0.05).

This indicates that while all bleaching agents affected the surface topography, the high‐concentration chemical agents in Opalescence Boost and Opalescence Home induced significantly greater roughness than the laser‐activated protocol, which maintained a surface profile statistically similar to that of the unbleached control group.

## 4 Discussion

Recently, minimally invasive dentistry has advanced due to improvements in materials. Over time, various antimicrobial and anti‐inflammatory agents have been proposed, thereby expanding the use of minimally invasive procedures to preserve natural tooth structure by developing more effective bioactive materials [7]. The Riva Star SDF consists of significant amounts of silver and fluoride as well as ammonia [Ag(NH_3_)_2_ F]. The reversible diamine‐silver ion complex [Ag(NH_3_)_2_], which is more stable than silver fluoride (AgF_2_), is formed by ammonia and silver ions. It can therefore be kept at a constant concentration over a longer period of time [11]. 38% SDF is colorless, highly alkaline (pH 12.5), and contains 5%–6% fluoride, 8.5%–10% ammonia, and 24%–27% silver [12]. However, a notable disadvantage of SDF is its tendency to cause significant dark discoloration of dentin, which limits its use, as well as the strong ammonia odor [20].

Similar to the Riva Star SDF, the Riva Star Aqua is a non‐invasive, two‐step treatment system. It is free of ammonia, thereby avoiding odor, an unpleasant taste, and gum irritation [29]. Unlike other Riva Star SDF formulations, Riva Star Aqua employs silver fluoride in its initial phase. Its silver and fluoride ions function similarly to those in the original Riva Star SDF, despite the absence of ammonia [29]. Silver ions exert antimicrobial effects by disrupting hydrogen bonds, inhibiting respiration, disrupting DNA, and interfering with cell division and cell wall synthesis [8].

A disadvantage of silver products is that they tend to stain demineralized areas of the tooth brown or black. Although this sometimes yields successful results, it also causes undesirable esthetic problems. Riva Star Aqua contains potassium iodide in the second step. While the formation of a silver iodide precipitate may reduce initial staining in some studies, recent systematic reviews indicate that the evidence regarding KI's efficacy in preventing long‐term discoloration is heterogeneous and inconsistent [24, 25].

Permanent human teeth were used in this study because they are large, readily available, and unique in radiodensity, enamel thickness, and dentin hardness. Salivary loading after applying Riva Star Aqua is crucial because it enables proper mineral absorption. This process involves calcium, phosphate, and fluoride forming fluorohydroxyapatite and calcium fluoride, which are essential for effectiveness.

Additionally, this loading is important for the darkening process, as phosphate reacts to produce silver phosphate within the tooth treated with Riva Star Aqua [21]. In this in vitro study, three bleaching protocols (Opalescence Boost (40% HP), Opalescence Home (15% HP), and Laser White 20) were evaluated for their effects on color change and enamel surface roughness of Riva Star Aqua‐treated remineralized caries‐like lesions under ideal conditions. According to studies, digital spectrometers are more accurate than colorimeters and visual detection techniques [39]. The VITA Easyshade spectrophotometer measures CIELab and identifies tooth shade using VITA 3D‐Master and VITA classical systems. It features a 20 W halogen lamp with a D65 illuminant and is cited in studies as the most accurate device for shade determination [40].

Surface roughness was measured in this study using profilometry, which provides precise, accurate results without requiring additional measurements. Research indicates that the profilometric method is an effective technique for quantitatively measuring surface roughness [41].

The primary objective of this in vitro study was to investigate the effects of different bleaching protocols on the color stability and surface roughness of enamel remineralized with a novel water‐based, ammonia‐free silver fluoride solution Riva Star Aqua.

Based on the findings, the first research hypothesis that the ammonia‐free silver fluoride solution would cause a clinically perceptible color change was accepted. The results demonstrated that the application of Riva Star Aqua caused significant discoloration in all specimens (Δ*E*_00_ > 1.8) [46], confirming that the ammonia‐free formulation still possesses the inherent staining potential associated with silver products.

The second research hypothesis stated that applying different bleaching protocols would significantly lighten enamel discoloration caused by the silver fluoride treatment. This hypothesis was also accepted, as all three tested bleaching protocols (Opalescence Home, Opalescence Boost, and Laser White 20) resulted in a statistically significant reduction in discoloration compared to the unbleached control group, effectively returning the enamel color closer to its baseline values.

Finally, the third research hypothesis that the application of bleaching protocols would significantly increase the surface roughness of the treated enamel was partially accepted. The profilometric analysis revealed that the Opalescence Home and Opalescence Boost bleaching protocols caused statistically significant increases in surface roughness (Ra) compared to their pre‐bleaching baseline. In contrast, the control group and Laser White 20 bleaching protocol showed increases in surface roughness (Ra) that were not statistically significant compared to their pre‐bleaching baseline. This confirms that while bleaching is effective for esthetic correction, it simultaneously alters the surface integrity of the remineralized enamel.

### 4.1 Color Stability (Δ*E*_00_ )

In this study, the bleaching effects of different bleaching agents on the enamel surfaces of teeth previously treated with Riva Star Aqua were investigated. The results show that although all bleaching agents produced a noticeable color difference (Δ*E*_00_ > 1.8) [46], there were significant differences in efficacy and effects on the enamel surface.

The Opalescence Boost group showed the most significant color difference (Δ*E*_00_ Bleach). This result contrasts with a previous study, in which better results were obtained with home bleaching (15% carbamide peroxide) than with in‐office systems (40% HP) in remineralized lesions [28]. This discrepancy could be due to differences in sample preparation, specific lesion characteristics, or the use of Riva Star Aqua in our methodology, which has influenced the diffusion and reactivity of the bleaching agents.

An important finding was that the control group showed the highest final color differences (Δ*E*_00_ Final), which suggests that the absence of bleaching either preserved some of the enamel's optical properties or that the silver‐free remineralization helped mask the color. Conversely, the Opalescence Boost group showed the lowest (Δ*E*_00_ Final). This could be due to interference from silver and fluoride deposits from silver sulfide‐based treatments, which are known to form a dense, less‐permeable layer on the enamel surface, limiting further oxidation and color change. The Laser White 20 group showed moderate results at all stages of color change.

The nearly similar Δ*E*_00_ Bleaching and Δ*E*_00_ Final values in the bleaching groups emphasize the importance of treatment duration and lesion composition in predicting bleaching outcomes.

Our results underline the complexity of bleaching lesions stained with silver fluoride. For instance, one study demonstrated that low‐concentration carbamide peroxide was effective in reducing discoloration caused by SDF in primary teeth. The use of KI potentially augments these esthetic outcomes [30]. However, we should be cautious when directly comparing our findings because of notable methodological differences. Unlike the previous study, which used primary enamel, our study employed permanent human enamel, which differs in thickness, permeability, and mineral density [30]. Furthermore, the presence of artificial saliva in our protocol—simulating the intraoral remineralizing environment—likely influenced the maturation and tenacity of the silver stain compared with studies in which this factor was absent. These variables (substrate type and environmental simulation) are critical limitations that may account for the varying degrees of bleaching efficacy observed across the literature. The results of our Opalescence Boost group confirm the findings of a study reporting that 35% hydrogen peroxide was the only agent with a bleaching effect on SDF‐treated artificial dentin caries [21]. This bleaching effect was most pronounced in the Opalescence Boost group, which demonstrates that regardless of bleaching agent concentration, a longer bleaching time for the enamel led to better color improvement [32].

One study also used 40% H_2_O_2_ as a bleaching agent after SDF treatment and observed a reduction in discoloration [22]. However, unlike our study, the samples were not exposed to artificial saliva, and the H_2_O_2_ was applied immediately after SDF application rather than after discoloration occurred [30]. This distinction is important because immediate bleaching yields different results than bleaching after the discoloration has fully set.

The intense discoloration after bleaching (Δ*E*_00_ Final) shows that removing the dark discoloration caused by Riva Star Aqua remains a significant challenge. This highlights the urgent need to develop innovative strategies and products that effectively treat dental caries with Riva Star Aqua, achieving a more esthetically satisfactory result.

### 4.2 Surface Roughness (Ra)

Before bleaching, the surface roughness values did not differ significantly between the groups, ensuring comparable initial conditions for the enamel. After bleaching, however, all test groups except Laser White 20 showed a statistically significant increase in surface roughness. Opalescence Boost showed the most substantial increase in roughness, closely followed by Opalescence Home, while Laser White 20 showed a slight, non‐significant increase. These results are consistent with previous studies indicating that bleaching leads to demineralization and erosion of the enamel surface [38], although the extent of these changes varies. The increase in roughness may be due to oxidative degradation of organic enamel components and partial mineral loss. As noted in some studies, the proximity of the bleaching agent to the tooth may cause microscopic changes on the enamel surface [34]. The reported effects of bleaching on enamel morphology are notably heterogeneous. While some studies report negligible alterations [36], others observe significant increases in roughness and porosity [37]. These discrepancies are likely driven by specific differences in commercial gel formulations rather than peroxide concentration alone. The pH of the bleaching agent is a critical factor; formulations with lower pH values have been shown to induce greater demineralization and surface alterations comparable to acid etching [31]. Additionally, the presence of specific desensitizing additives, such as fluoride or potassium nitrate, and the type of thickening agents used modulate the demineralization process, potentially protecting the enamel surface and accounting for the varying results observed in the literature.

The mean roughness profile of Laser White 20 suggests that it provides a good balance between esthetic enhancement and enamel preservation. In contrast, the two Opalescence products, particularly the Opalescence Home system, caused more significant surface changes. This impacts the long‐term health of the enamel and requires subsequent remineralization treatments to mitigate the increased roughness.

### 4.3 Clinical Relevance

These findings suggest clinicians should carefully consider bleaching protocols after caries treatment with ammonia‐free silver fluoride. Highly concentrated peroxide speed bleaching but risks increasing enamel roughness and demineralization, as confirmed by recent analyses [35]. In contrast, laser‐activated bleaching has proven to be effective, with a more conservative effect on enamel and controlled intrapulpal temperatures within safe limits. This aspect (intrapulpal temperatures) was not evaluated in this study, but it is often considered an advantage of laser‐activated bleaching in clinical settings [44, 45]. However, these in vitro findings should be interpreted cautiously. A systematic review and meta‐analysis found that light or laser activation does not consistently improve the efficacy of in‐office bleaching over chemical activation alone and may increase the risk of tooth sensitivity [33]. While the laser protocol was compatible with the silver‐fluoride surface in this study, clinicians should consider the lack of consensus on its added benefit versus the potential increased cost and sensitivity. A trial comparing in‐office bleaching with 18%, 25%, and 40% hydrogen peroxide showed similar effects. Tooth sensitivity increased immediately but returned to baseline within a week, even at the highest concentration. Short‐term sensitivity is common. Reviews show that peroxide concentration, application time, and gel properties influence the risk of sensitivity. This study didn't evaluate patient sensitivity, but it's clinically relevant. Future research should include sensitivity to guide treatment after silver fluoride.

### 4.4 Limitations of This Study

This in vitro model provides useful insights but has limitations in generalizability. The demineralization protocol using continuous acetic acid at pH 4.4 mimics erosion rather than natural caries. While standardized, it lacks the pH cycling and biofilm interactions of real cariogenic environments, which influence lesion depth, mineral composition, and fluoride penetration. It also does not include a dynamic biofilm challenge or salivary flow, both of which are essential to oral demineralization‐remineralization cycles. Therefore, interactions with bleaching agents and silver fluoride may vary clinically due to pellicle and bacterial presence. Additionally, although this study used permanent human teeth, silver fluoride treatments are recommended for both permanent and primary teeth. Variations in enamel thickness, permeability, and prism structure mean that the silver penetration and bleaching effectiveness observed here may not directly apply to pediatric teeth. The lack of a traditional SDF‐positive control group is a limitation, and we strongly recommend including this comparison in future research.

## 5 Conclusion

Within the limitations of this in vitro study, it was concluded that:

1. The application of the water‐based, ammonia‐free silver fluoride solution (Riva Star Aqua) resulted in a significant and clinically perceptible discoloration of the remineralized enamel.
2. All three tested bleaching protocols (Opalescence Home, Opalescence Boost, and Laser White 20) were effective in significantly lightening the discoloration caused by the silver fluoride treatment.
3. Regarding color efficacy, no statistically significant differences were found among the three active bleaching protocols; all achieved a comparable final esthetic outcome.
4. Regarding surface integrity, all bleaching protocols resulted in a significant increase in roughness relative to the pre‐bleaching baseline. However, the laser‐activated protocol Laser White 20 resulted in significantly lower final roughness values than those of the conventional in‐office and at‐home bleaching agents, while maintaining a surface profile statistically comparable to that of the unbleached control group.

## Funding

The authors have nothing to report.

## Disclosure

The authors have nothing to report.

## Conflicts of Interest

The authors declare no conflicts of interest.

## Data Availability

The data that support the findings of this study are available from the corresponding author upon reasonable request.
